# Randomized clinical trial to evaluate two methods of caries risk assessment in schoolchildren: the CARDEC-PEL 04 study protocol

**DOI:** 10.1186/s12903-021-02010-3

**Published:** 2021-12-18

**Authors:** Aryane Marques Menegaz, Thays Torres do Vale Oliveira, Mariana Minatel Braga, Daniela Prócida Raggio, Maximiliano Sergio Cenci, Fausto Medeiros Mendes, Marina Sousa Azevedo

**Affiliations:** 1 Graduate Program in Dentistry, Federal University of Pelotas, Pelotas, Brazil; 2 Department of Pediatric Dentistry, School of Dentistry, University of São Paulo, São Paulo, Brazil

**Keywords:** Dental caries, Randomized clinical trial, Children, Risk assessment

## Abstract

**Background:**

Caries risk assessment is an essential element for managing and preventing dental caries in children. Individual caries risk assessment can be conducted to evaluate the presence or absence of single factors, or using multivariate models, a combination of factors. The subject has been extensively studied, but no previous research has compared whether a more elaborate and individualized method of caries risk benefits the patient than more straightforward strategies. Thus, this protocol evaluates the efficacy of two risk assessment methods for caries control in children, a simplified method based on caries experience evaluation and a multivariate method described in the literature.

**Methods:**

This is a randomized, double-blind, controlled, parallel-treatment trial protocol. Two groups will be tested for two forms of caries risk assessment: an individualized and detailed multivariate method based on the guidelines of the Caries Care International 4D and another simplified process, based only on caries experience in primary and/or permanent dentition, considering the presence of decayed, missing and filled teeth using the DMFT/dmft index. Participants will be children aged 8 to 11 years, followed up at 12 and 24 months. The primary outcome will be a composite outcome representing the number of tooth surfaces requiring operative intervention (account variable). In addition, the Shapiro–Wilk normality test and Student's t-test will be performed. A multivariate analysis using negative binomial regression will compare groups in the intention-to-treat population, considering a two-tailed significance level of 5%.

**Discussion:**

This is the first randomized clinical trial aiming to compare dental caries-related treatment and follow-up based on a detailed, multivariate and individualized assessment of caries risk in school-age children to a simpler risk assessment strategy based on caries experience. This study will define whether there are essential benefits to the patient that justify the choice of one method over the other.

*Trial registration* Clinicaltrials.gov registration: NCT03969628. Registered on May 31th, 2019.

**Supplementary Information:**

The online version contains supplementary material available at 10.1186/s12903-021-02010-3.

## Introduction

Dental caries is considered a multifactorial dynamic disease, determined by the interaction of biological, behavioral, and psychosocial factors related to the individual's environment [1]. There has been a continuous decline in the caries prevalence and lesions progression in the last decades [2]. However, unequal distribution of the disease burden in the population is still observed, and poorer children with low access to dental services are more affected [3].

Authors have stated that caries risk assessment can prevent the onset of the disease or its evolution and, thus, could contribute to guide public interventions and allocate time and resources to those most needed [4]. In addition to being considered an essential element for management and prevention of dental caries, caries risk assessment, could establish the appropriate follow-up appointments [5–7].

The main goals of caries risk assessment are determining the patient's probability of developing new caries lesions in a given period and the risk of present lesions progressing to more severe stages [5, 6]. There are mainly two ways to assess individual caries risk: (a) simple assessment based on factor’s presence or absence, and (b) assessment by multivariate models, a combination of more than one variable, classifying the patient’s caries risk [6, 8].

Several studies about caries risk assessment with children and adolescents were carried out and are summarized in systematic reviews [8–11]. Most primary studies have cohort design and were conducted to determine isolated variables that would have adequate predictive power for developing new carious lesions [12]. Those were unanimous in stating that the past caries experience is the factor with the strongest association with an increased risk of dental caries. In the cohort study by Hall-Scullin, et al., they show that children who had caries lesions in the primary dentition and those who were caries-free at this stage had very different trajectories in the development of new caries lesions in the permanent dentition; hence the authors suggest different prevention strategies for these two groups of children [12].

However, other authors claim that past caries experience would not be the ideal prediction method, especially in the new era in which dentistry is of minimal intervention and non-operative approaches concerning the management of dental caries [5]. Multivariate methods have been proposed to improve this prediction power [5, 6, 8, 11, 13]. Some authors claim that multivariate models have better predictive power than individual clinical indicators, especially preschool children [6, 7]. However, to date, there is no scientific evidence that supports choosing one factor or method over another, and none are very accurate in predicting the development of new lesions. At most, they can be considered having moderate evidence level [10, 14].

Although authors and clinical protocol guides have emphasized the importance of individualized caries risk assessment for the correct management and non-operative treatment of patients [4, 7, 13, 15], this assumption is mainly based on expert’s opinion. Furthermore, the studies on which the guides are based are of low methodological quality [14], and are focused on identifying the factors or models that predict new injuries.

No study has been carried out to assess whether a more elaborate and individualized method of caries risk assessment, considering a combination of several factors, provides more effective prevention and treatment related to dental caries. Moreover, there is no information regarding potential benefit to the patient, compared to more straightforward strategies, as caries experience in primary and/or permanent dentition. This would ideally be answered through a randomized clinical trial comparing the two strategies.

Thus, a randomized clinical trial will be conducted to evaluate two methods of assessing children’s caries risk. This protocol will compare dental caries-related treatment and follow-up based on a detailed, multivariate and individualized assessment of caries risk in children to a more uncomplicated risk assessment strategy based on caries experience.

## Methods

### Trial design

This is a randomized, double-blind, controlled, parallel trial-treatment trial. Two groups will be tested regarding caries risk assessment: individualized and detailed method, considering several risk predictors, based on the guidelines of the Caries Care International 4D [4, 5, 16], and another simplified method, based only on the caries experience in deciduous and/or permanent dentition, considering the index of decayed, missing and filled teeth (DMFT/dmft) [12]. The groups will receive treatment and follow-up procedures based on their form of caries risk assessment.

The study was registered in the ClinicalTrials.gov clinical database (Identifier: NCT03969628), approved by the Research Ethics Committee from the School of Dentistry, Federal University of Pelotas (UFPel) (#3.282.962) and reported following the guidelines of the “Standard Protocol Items: Recommendations for Interventional Trials” (SPIRIT), detailed in the Additional file 1.

### Participants, intervention, and outcomes

#### Setting

The study will be conducted at the Pediatric Dentistry Clinical of Dental School, Federal University of Pelotas (UFPel). Children from 8 to 11 years old who seek assistance at the location will be eligible, after duly authorized by the guardians, through free informed consent and the child's consent.

#### Eligibility: inclusion and exclusion criteria

The study will include children who meet the following criteria: (1) 8 to 11 years old, (2) who were referred or seeking dental treatment at the college and have been screened by the Pediatric Dentistry Clinical, (3) residents in the city from Pelotas/RS or nearby region (not exceeding 50 km of distance). In addition, children will be excluded: (1) wearing fixed appliances, (2) with systemic problems or with any disability that lead to difficulty in understanding the guidelines and the questionnaire, (3) with a history of absences (up to 2 attempts to schedule an interview and exam), (4) with an expected change of city and region (cities 50 km far from Pelotas), (5) manifesting behavioral problems already in the screening clinical appointment, specifically, that present the classification “generalized protest” or “more intense protest” according to the Brazilian Version of the VENHAM Scale (BvVBRS). They will be referred to behavior management consultation [17].

#### Intervention

Two ways of assessing the risk of dental caries will be tested so that this study will have two intervention arms:

Multivariate Group: Children classified as low and high risk for caries and treated based on the Caries Care International 4D manual [15, 16], in which different caries risk profiles are directed to different strategies for caries management.

Simplified Group: Children classified as low and high risk for caries based only on past and current caries experience in primary and/or permanent dentition and treated according to that [12].

**Fig. 1:**
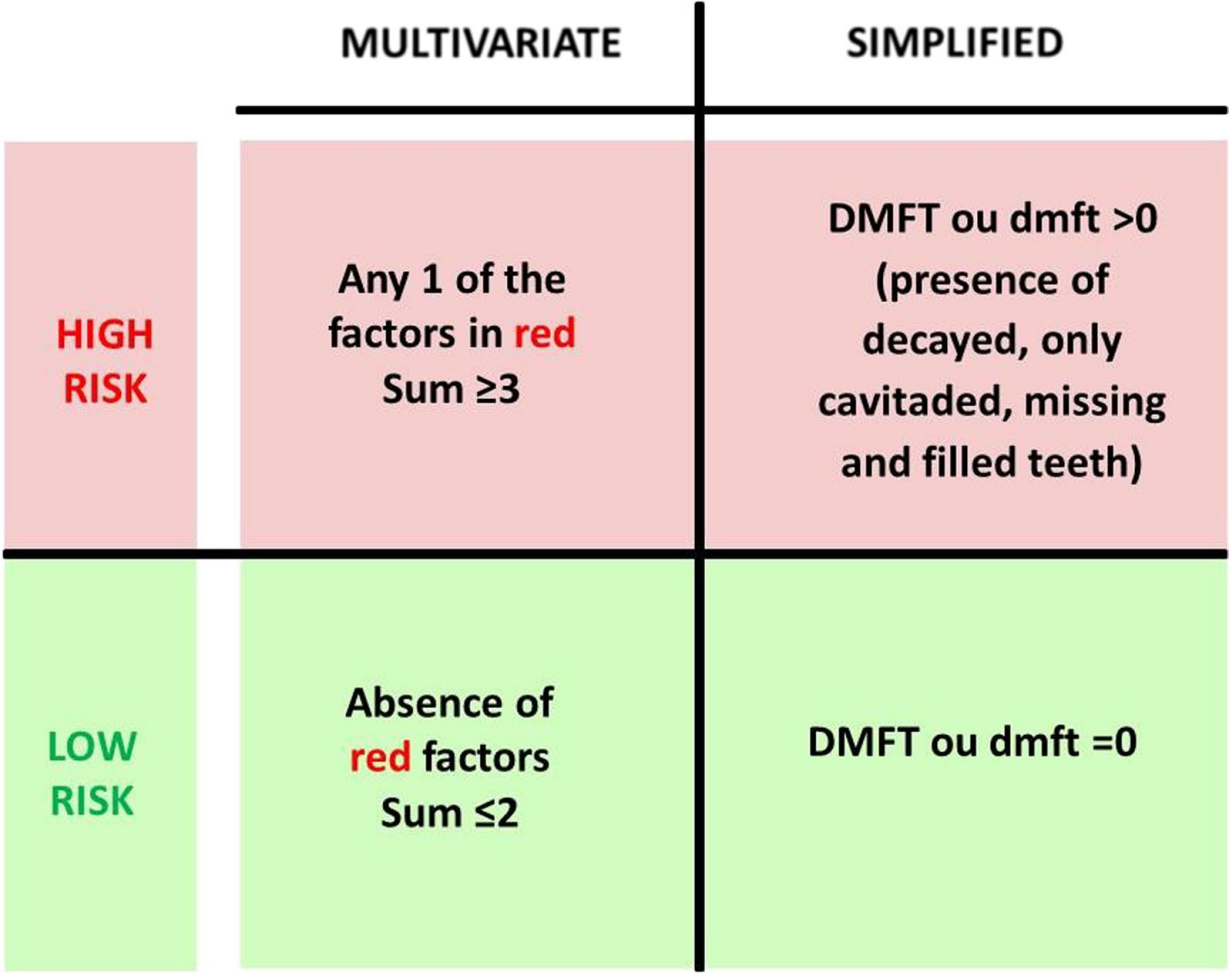
Classification of caries risk according to the criteria

The Caries Care International 4D are a series of clinical protocols for diagnosis and treatment decisions related to non-operative and operative procedures aimed to maintaining health and preserving tooth structure [16]. One of the key elements is the individual assessment of the patients' caries risk, which leads to a treatment based on prevention, control, and minimally invasive surgical treatment personalized according to the needs of each patient, as well as a follow-up with intervals based on the initial risk [4, 15]. The individualized and detailed method is based on several risk predictors, and its latest update proposes the classification of the patient as low and high risk [16] (Fig. 1).

The risk assessment based only on caries experience in primary and/or permanent dentition, considering decayed, missing, or filled teeth by caries, according to the criteria of the World Health Organization (WHO) [16], will be the criterion choice of the simplified group. Thus, children will be classified as low, with no past or current caries experience (DMFT/dmft = 0) and high risk, with history or current caries experience in primary and/or permanent dentition (DMFT/dmft > 0) and will be treated and followed-up based on clinical caries exam [12] (Fig. 1).

**Fig. 2:**
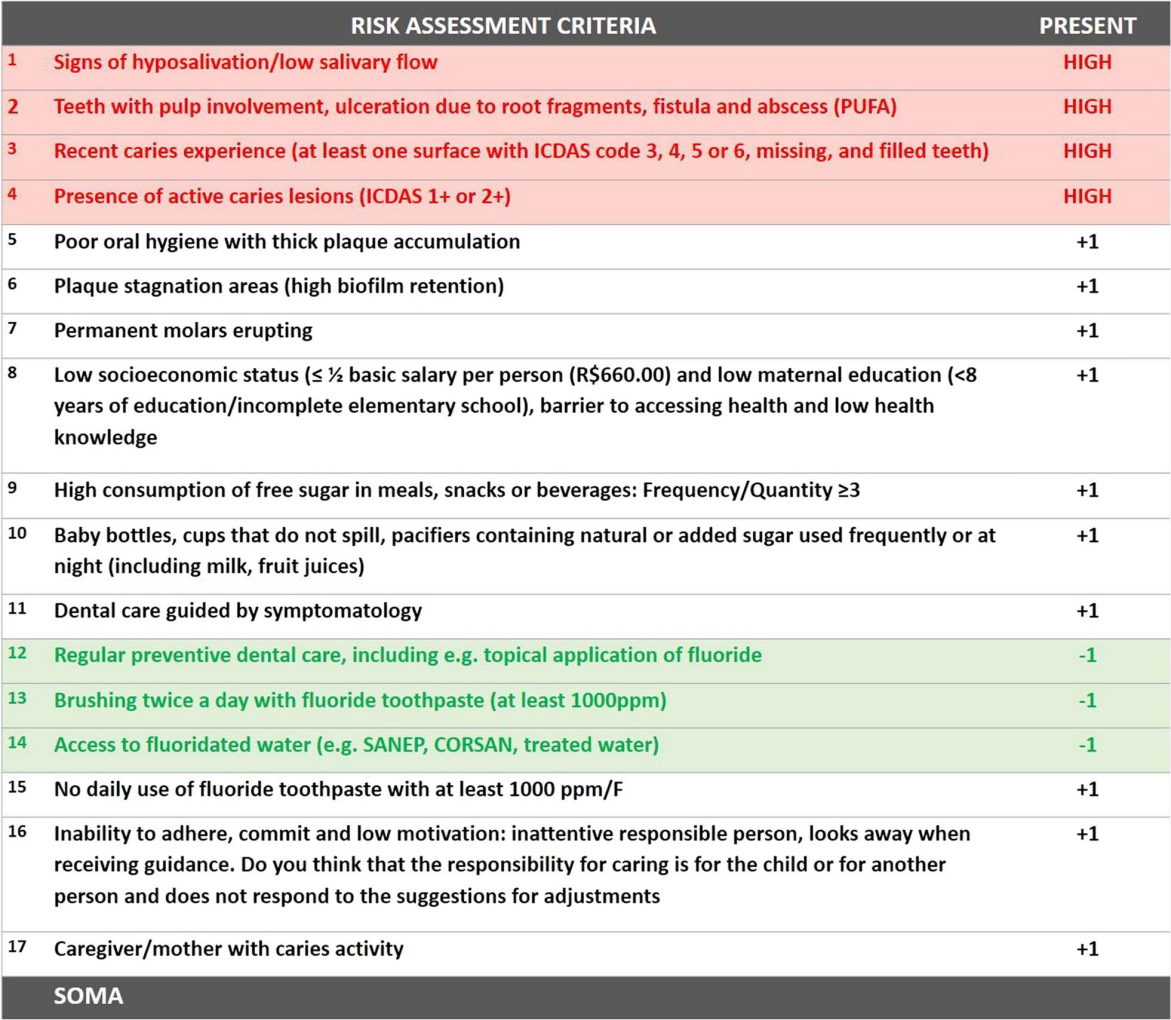
Simplified and multivariate caries risk assessment criteria

The risk assessment criteria are shown in Fig. 2. For the Multivariate group, a practical method of association of caries risk factors and protective factors will be used, represented by colors: red and black for risk factors, green for the protective factors. The patient will be evaluated according to their presence or absence, resulting in a final sum that will classify the child as high or low risk. For example, a patient with any risk factor (red color) is considered high risk. Those with risk factors (black color) receive 1 positive point for each factor present. Those with caries protective factors receive 1 negative point. If the patient does not have risk factors in red, which already puts him at high risk regardless of the score, the risk of final caries will be given through a combination based on a summation of risk factors (+) (black color) and protective (−) (green color). Having a combination of factors with a final sum ≥ 3 classifies the patient as high risk. Patients classified as low risk are those with a final sum ≤ 2 [16].

#### Interview, examination procedures, and treatment of participants

The child legal guardian will answer a semi-structured questionnaire regarding data needed for the risk assessment criteria listed in Fig. 2. Demographic and socioeconomic data will be collected, such as: sex, age, income, parents' education, and skin color. In addition to the standard questions aimed at providing comprehensive care to the child, the caregiver will be asked about the diet to identify sugar consumption and oral hygiene (regularity and use of fluoride toothpaste). This interview will address specific questions included in the risk assessment (criteria 8–16) (Fig. 2).

The clinical examination will follow a sequence, initially the absence or presence of: thick dental plaque, areas of plaque stagnation (high biofilm retention), and erupting molars will be evaluated. Afterward, a trained and calibrated evaluator will assess the condition for dental caries according to the WHO criteria through the DMFT/dmft index (decayed, missing, and filled teeth) [16]. Then, a professional toothbrushing will be carried out with fluoride toothpaste, and the same trained and calibrated evaluator will assess caries using the International Caries Detection and Assessment System (ICDAS) index in its simplified form [16]. Caries activity will be analyzed using the visual criteria proposed by the NYVAD System and finally the complementary PUFA/pufa index (pulp involvement, ulceration due to root fragments, fistula and abscess for permanent and primary teeth) [16].

**Fig. 3:**
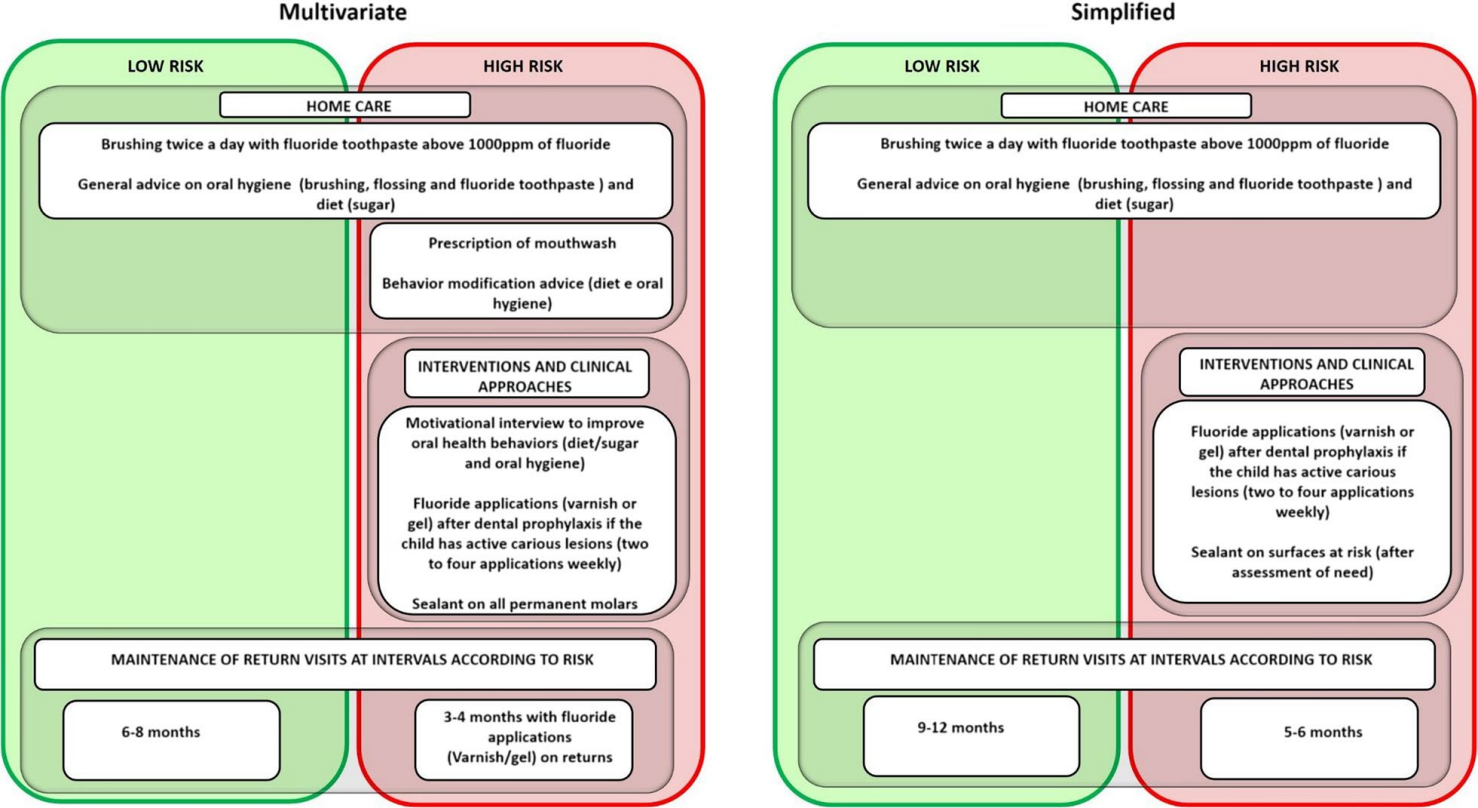
Caries risk guide and clinical management

After the interview and clinical examination, the researcher will conduct the caries risk assessment expressed in Fig. 1, using the criteria proposed by Caries Care International 4D [15, 16], together with caries experience in primary and/or permanent dentition. The mother's caries experience will be evaluated using the DMFT index only if the score referring to the mother's exam can change the classification; this will be the last item to be listed [16]. The participant will be classified using both methods: simplified and multivariate, by the same researcher. Only after complete classification they will be randomized into one of the groups and follow the intervention and subsequent clinical procedures according to their group, shown in Fig. 3.

The multivariate group follows the protocols proposed by the Caries Care International 4D that guide prevention strategies and early management of dental caries. General advice on diet and oral hygiene are common for high and low-risk patients (brushing twice a day with fluoride toothpaste above 1000 ppm of fluoride). Patients classified at high risk are recommended to be approached with motivational interview techniques for behavior modification, prescription of mouthwash as one of the home care and clinical approaches including: fluoride applications (varnish or gel) after dental prophylaxis if the child has active carious lesions (two to four applications weekly) and sealant on all permanent molars. The scheduled return intervals for low-risk patients are six to eight months and high-risk patients three to four months. In these high-risk patients, the application of fluor gel or varnish is recommended at each visit.

For the simplified group, care protocols that are a consensus in clinical practice were used, following the current routine in the unit (Dental School—UFPel). Both patients classified as high and low risk received general advice on diet and oral hygiene (brushing twice a day with fluoride toothpaste above 1000 ppm fluoride). Among the interventions and clinical approaches unique to high-risk patients in the simplified group are: sealing permanent molars on surfaces classified as at risk after evaluation, for example, molars with deep grooves and retention factors. And the fluoride applications (varnish or gel) after dental prophylaxis if the child has active caries lesions (two to four applications with a weekly interval). The maintenance of the return visits has the interval: patients classified as low risk nine to twelve months, high-risk patients five to six months the returns.

### Randomization, implementation, and blinding

The randomization strategy used will be in permuted blocks with sizes of 4, 6 and 8 and stratified by age (8 to 9 years; 10 to 11 years) and the child's caries experience (DMFS/dmfs ≤ 4; DMFS/dmfs > 4). First, all participants will be evaluated for caries risk by the two strategies, and then participant randomization will be performed in the same session. The allocation sequence used will be obtained from the website www.sealedenvelope.com, and sealed in sequentially numbered opaque envelopes by a researcher without any contact with the practical research procedures.

Another researcher will be responsible for including the participants. Although the groups to which children will be allocated will not be revealed to children and their parents, due to differences in treatments and mainly different intervals for return visits in the two groups, it is difficult to maintain blinding throughout the study for all participants. Therefore, the outcome assessors at 12 and 24 months will be blinded to groups. The groups will be coded in the data analysis worksheets for blinding those who will carry out the statistical analysis. Breaking blinding will not be allowed under any circumstances for outcome evaluators.

### Follow-up visits

Participants will return at intervals determined by the patient's individual risk, according to groups. These appointments were considered as part of the allocated intervention and will follow clinical management protocols according to your group. After 12 and 24 months they will also return to collect the outcomes. A new risk assessment will be performed, following the protocol to which the child was initially allocated, as well as a further clinical examination that will reassess: plaque dental caries, condition, and activity through the DMFT/dmft, ICDAS, NYVAD, and PUFA complementary index [18, 19]. The researcher responsible for the inclusion will carry out this reassessment, and if necessary, a new treatment plan will be developed. Two trained and calibrated examiners, blind in relation to the groups to which the participants were allocated and without participation in the dental treatments, will re-examine the children. After the re-examination, the child will follow the standard re-evaluation procedure described above and will be referred for treatment, if necessary.

### Outcomes

The primary outcome will be a composite outcome representing the number of tooth surfaces requiring operative intervention in two years of follow-up. Thus, the primary outcome will be the sum of surfaces with new carious lesions involving dentin, restored surfaces that needed replacement, and those teeth needing endodontic treatment or extraction (accounting for 4 or 5 surfaces per tooth, for anterior or posterior teeth, respectively). If the tooth surface requires two types of treatment at different times, the treatment will be computed twice.

Secondary outcomes will be the components of the primary outcome considered separately: surfaces with new lesions involving dentin, restored surfaces in need of restoration replacement, teeth with episodes of pain, need of endodontic treatment and extraction. Besides that, costs accumulated for 24 months, health-related quality of life (HRQOL), and oral health-related quality of life (OHRQOL) will also be collected as secondary outcomes.

In order to assess the cost of going to the appointments during the 2 years of follow-up questions about travel will be collected: “What transport do you normally use to come to the appointments?”; “How many buses do you take to come?—If the person uses a bus.”; and “How long does it take, approximately, from leaving home until arriving at the dental school?”.

The HRQOL will be collected using the Brazilian version of the KIDSCREEN-52 [20] and for OHRQOL the Brazilian version of the Child Perceptions Questionnaire (CPQ) [21] will be used. The questionnaires will be applied by trained interviewers before the clinical examination.

For the economic evaluation, a specific analysis plan of economic economic will be prepared. The main economic hypothesis will be if using the multivariate (more complex) assessment may be an efficient manner of allocating resources. A societal perspective and a 24-month time horizon were adopted. Resources spent during the trial follow-up will be registered and then, appropriately valued as costs. The effects to be tested will be derived from the assessed outcomes: number of new operative interventions alongside 24 months, changes in DMFT after 24 months and Quality of life. Then, cost-effectiveness analyses will be performed.

### Timeline

**Fig. 4:**
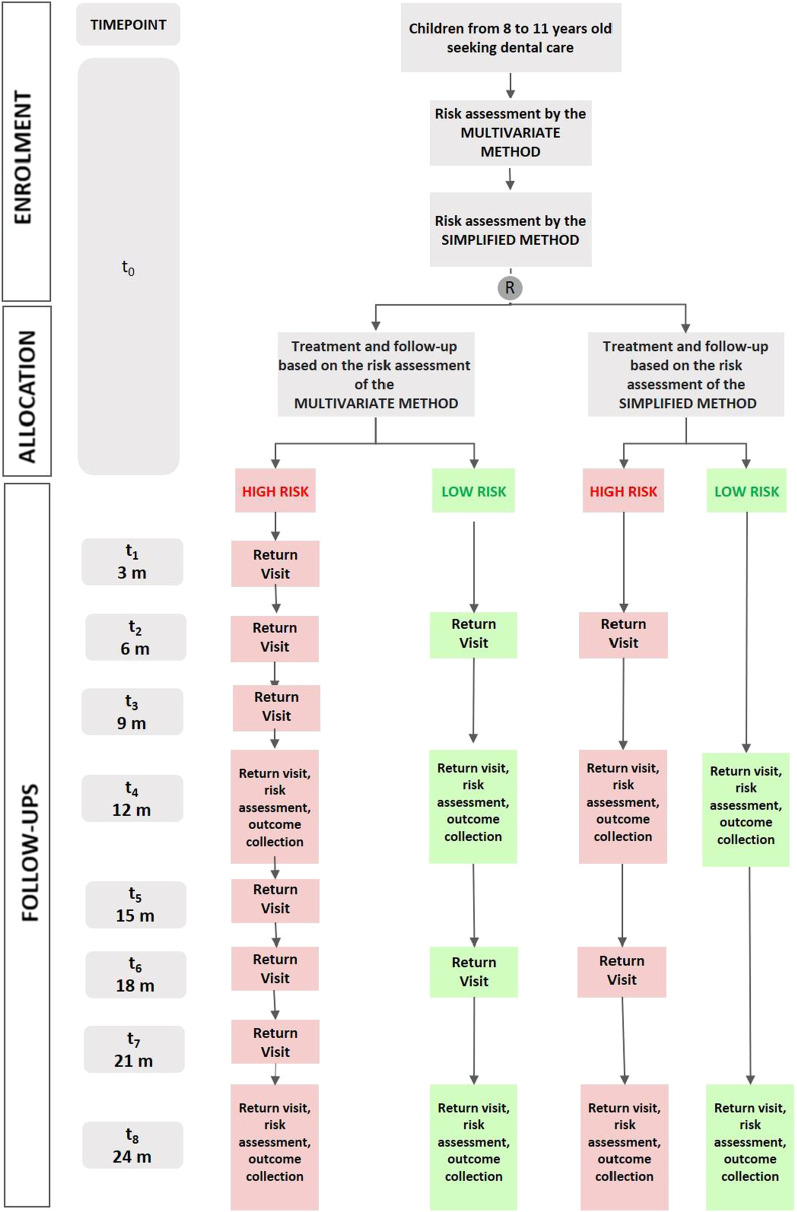
Standard protocol items timeline, enrolment, registration, feedback, and evaluations

The participant will be followed for 24 months, at 12 and 24 months, return consultations will be held for risk assessment and outcome collection. The other appointments will be according to caries risk assessment. The study phases are described in Fig. 4.

### Sample size

The sample estimation was based on the primary outcome, which is the number of tooth surfaces in need of surgery during follow-up. Therefore, the following possibilities were considered to compose this outcome:

- New caries lesions;
- Restorations in need of replacement;
- Dental extractions (5 surfaces per tooth);
- Resolution of pain episodes and/or need for endodontic treatment (5 surfaces per tooth).

To perform the sample calculation, incidence data were collected for all possibilities in the literature, reaching an average occurrence of 17.6 new decayed surfaces in 2 years [22], 10% failure of occlusal-proximal and composite resin and glass ionomer occlusal restorations, which gives an average of 0.1 surface with the need to change for occlusal restorations and 0.2 surfaces in 2 years [23], 0.08 tooth extractions for caries in two years, totaling 0.2 surfaces [24] and 0.2 pain episodes in 2 years on average, leading to an average of 1 surface treated in 2 years. Thus, the estimate of surfaces in need of surgical treatment in 2 years is an average of 19. It was considered that the reduction of 5 surfaces in need of treatment would be a significant number. The expected mean for the test group would be 14. The expected standard deviation values for the control and test groups are 15 and 10, respectively [22–24]. Considering a significance level of 0.05 and a power of 0.80, using a two-tailed test, the minimum number of children per group calculated was 103. Considering a sample loss of 20%, the final rounded number required of 250 children was reached.

### Recruitment

Recruitment will occur at the Dental School, a reference place for dental treatment in children, and receives many patients who seek care or are referred from other units. Approximately 600 patients receive treatment per year. Participants will be randomly selected from this broad sample.

### Data collection, management and analysis

#### Data collection

A trained and calibrated (weighted Kappa value greater than 0.80) examiner will perform the initial exams. Periodic checks (every 50 patients included in the study) of the calibration will be made in 10% of the sample.

Another evaluator, who has not participated in any previous phase of the study, will reassess the participants for the collection of outcomes after 12 and 24 months. This examiner will also be trained and calibrated before the start of follow-ups and periodically for every 50 evaluated participants. An intraclass correlation coefficient greater than 0.80 must be obtained before starting the assessments.

Regarding the collection of outcomes, the examiners will evaluate the children regarding: (1) the number of dental surfaces with decay in need of operative treatment, with dentinal involvement, cavitated or not; (2) the number of restored surfaces that need replacement (extensive failures, carious lesions around the restorations or complete loss of restorative material); (3) restorations in need of repair; (4) teeth with episodes of pain, need for endodontic treatment or extraction.

#### Data management

The groups will be coded in the analysis and data will be transferred to spreadsheets after the different phases of the study (baseline, 12 months, and 24 months). An external researcher will check the data for missing data, out-of-range values, and illogical or invalid responses weekly. In case of detection of protocol deviations, immediate solution may be established for guarantee the trial quality. In case of missed (or incorrect) data, data imputations may be indicated. After the publication of the main results, anonymous data will be shared in a public repository.

#### Data analysis

Outcomes will be analyzed as count variables. Shapiro–Wilk normality test will be performed. If it presents normal distribution, to compare the primary outcome between the two groups, Student's t-test will be performed. Regarding secondary outcomes, these will also be analyzed by Student's t-test if they have a normal distribution.

A multivariate analysis using negative binomial regression will also be used to compare groups, adjusting for socioeconomic and demographic variables (gender, age, number of children, family structure, and income). Two-tailed tests will be used for all analyses, and the significance level will be set at 5%.

### Monitoring

#### Data monitoring

Data collection, management, control and analysis will be undertaken independently by MSA.

#### Harms

Possible adverse effects are inherent to dental treatments in general and are already managed by the care unit as a clinical routine. Therefore, the study does not pose a risk to the oral health of participants. If any harm related to the proposed intervention in one of the groups is clearly detected, the study may be suspended and participants will receive the best option of intervention available.

#### Auditing

The data entered will be managed by the study authors. Data will be reviewed periodically. Inconsistencies will be checked, corrected, and recorded.

### Ethics and dissemination

#### Research ethics approval

This study was submitted and approved by the Research Ethics Committee of the Dental School, Federal University of Pelotas (UFPel) (#3.282.962).

#### Consent and assent

Patients will be duly authorized by the guardians, through informed consent and the consent of the child.

#### Confidentiality

Identification numbers will be used to guarantee the participant's confidentiality during data analysis. In addition, participant files will be stored in a secure location.

#### Availability of data

The study data used will be available from the corresponding author (MSA) upon reasonable request.

#### Ancillary care and post-research

Participants will receive dental treatment during and after the end of the study.

#### Dissemination policy

The results will be reported in full through national and international journals, websites, and patient newsletters. As well as disseminating the results to public bodies, to eventually guide public policies and/or inclusion in guidelines.

## Discussion

The risk assessment for dental caries is still a challenge for Dentistry. It becomes even more complex in Pediatric Dentistry, as the child is in constant growth and development. The lack of well-designed studies leads to a series of caries risk proposals, based on one or several criteria, with different assessment systems. This heterogeneity of methods confuses dental professionals and makes the decision-making process difficult.

It is known that the dynamic and multifactorial process of caries disease has led researchers to consider important the risk assessment by multivariate methods, whether in the paper form model or computerized assessment. Standard tools are Cariogram, ADA, CAMBRA, and ICCMS™; many uses three or more caries risk categories. The Cariogram, an open-access software that refers to the probability of developing new caries lesions was used in the majority of well-designed studies. Still, the program's accuracy for children is limited, and its applicability becomes dependent on the use of computers, which is not a reality in different populations, making its generalization restricted [8].

The *Caries Care International* was developed to promote a patient-centered risk-based approach to dental caries management designed for the clinical dental practice, shares the same goals as the ICCMS™ system intended to help patients and dentists control the caries process and maintain health [15]. The four interlinked steps in the cycle all start with a 'D': 1st D: Determine the risk of caries; 2nd D: Detect injuries, scale their severity and assess their activity status; 3rd D: Decide on the most appropriate care plan for the patient at that time; 4th D: Do—implementation of the treatment plan always seeking to preserve the dental structure and control of the patient. Therefore, they are referred to in Caries Care International as the four Ds. Caries risk assessment is the essential first step in the 4D cycle, personalized method that features two risk categories, low risk and high risk [16].

There is a consensus that caries experience is the most obvious factor in predicting new caries lesions. Therefore, its application in older children would be indicated. Although it is considered an easy-to-apply and straightforward method, the accuracy of this indicator is still moderate. Thus, age is an essential factor to be considered. In younger children and preschoolers, the presence or absence of caries is not effective, as there was no time for the disease to manifest. In these cases, the multivariate models may have a more suitable predictive value [9]. As it is a very comprehensive guide, the applicability of Caries Care International in clinical practice is very time-consuming and challenging for the clinician to understand. Developing a practical guide considering the relevant risk factors for the specific group of children would be an important contribution.

The motivation for this study is that it would be essential to establish safer and more effective parameters for this theme and test different methods (simplified and multivariate) to understand whether there is a difference between these two approaches. In addition, developing a well-designed randomized clinical trial, considered solid scientific evidence, is necessary to answer this research question relevant to clinical practice, but it still has several gaps.

The new calls of scheduled returns for each group were organized with a flexibility interval of around two months; for the multivariate group, the returns for low-risk patients are six to eight months and high-risk patients three to four months, and for the simplified group patients classified as low risk, returns are from nine to twelve months and high-risk patients from five to six months. It is considered an important alternative that enables the protocol's applicability in various scenarios with different logistics of appointments.

Among the limitations of this study, we highlight the sample, a restricted population with specific characteristics, high present or past experience of caries, who seek care or are referred to the service that is a reference in clinical pediatric care in the locality. Although the treatments will be carried out in the same place, a clinical unit, the contamination of information and experiences can be a reality among the patients' families. Nevertheless, it is believed that these possible limitations do not directly interfere with the course of the study, being moderated by the coordinating team of the study.

The hypothesis is that both methods present very similar and coincident results. Thus, parameters such as the patient's and dentist's vision, workplace setting can be decisive in the choice, the clinician could choose the one that best suits their practice and is readily applicable to their reality and that of his patient.

### Study status

Suspended: Study halted prematurely because of 2019 Novel Coronavirus (COVID-19) pandemic but potentially will resume.

## Supplementary Information

**Additional file 1.** SPIRIT Checklist: Recommended items to address in a clinical trial protocol and related documents.

## Data Availability

The datasets of the study may be obtained from corresponding author upon reasonable request, as long as it does not violate patient confidentiality.

## Abbreviations

CARDEC: Caries detection in children
DMFT/dmft: Decayed, missing, filled, permanent or deciduous teeth
SPIRIT: Standard protocol items for clinical trials
UFPEL: Federal University of Pelotas
WHO: World Health Organization
FDI: World Dental Dederation
ICCMS: International Caries Classification and Management System
ICDAS: Caries Detection and Assessment System
PUFA: Pulp involvement, ulceration due to root fragments, fistula and abscess for permanent and primary teeth
CPQ: Child Perceptions Questionnaire
